# Precision dosing of voriconazole in immunocompromised children under 2 years: integrated machine learning and population pharmacokinetic modeling

**DOI:** 10.3389/fphar.2025.1671652

**Published:** 2025-09-15

**Authors:** Li Shen, Mengdi Hu, Xiaoyong Xu, Yuxuan Zhou, Wei Wu, Xilin Ge, Guangfei Wang, Yi Wang, Zhiping Li

**Affiliations:** ^1^ Department of Clinical Pharmacy, Children’s Hospital of Fudan University, National Children’s Medical Center, Shanghai, China; ^2^ Department of Pharmacy, Suzhou Hospital, Affiliated Hospital of Medical School, Nanjing University, Suzhou, Jiangsu, China; ^3^ Global Health Research Center, Duke Kunshan University, Kunshan, Jiangsu, China; ^4^ Department of Pharmacy, The First Affiliated Hospital of Fujian Medical University, Fuzhou, China; ^5^ Department of Pharmacy, National Regional Medical Center, Binhai Campus of the First Affiliated Hospital, Fujian Medical University, Fuzhou, China; ^6^ Department of Neurology, Children’s Hospital of Fudan University, National Children’s Medical Center, Shanghai, China; ^7^ Department of Pharmacy, Kunshan Women and Children’s Healthcare Hospital, Children’s Hospital of Fudan University, Kunshan Branch, Kunshan, Jiangsu, China

**Keywords:** voriconazole, children, under 2 years, machine learning, SHAP analysis

## Abstract

**Objective:**

This study aimed to develop an individualized dosing strategy for voriconazole (VRZ) in children under 2 years of age by integrating machine learning (ML) and population pharmacokinetic (PopPK) modeling.

**Methods:**

This retrospective observational study included 76 eligible pediatric patients for model development, analyzing their baseline characteristics and laboratory parameters. A population pharmacokinetic (PopPK) model using NONMEM^®^ software was performed to assess the clearance (CL) and volume of distribution (V) of VRZ. The individual CL and V were included as input variables. The Boruta algorithm was employed for feature selection, after which six machine learning algorithms were applied. The models were evaluated using Mean Squared Error (MSE), Root Mean Squared Error (RMSE), Mean Absolute Error (MAE), and coefficient of determination (R^2^) to identify the optimal algorithm, which then underwent independent external validation. The selected final model was analyzed for interpretability using Shapley Additive Explanations (SHAP).

**Results:**

A total of 76 pediatric patients were enrolled for model development, consisting of 58 males (76.3%) and 18 females (23.7%), with a median age of 11 months and a median weight of 8.05 kg. We analyzed 110 therapeutic drug monitoring (TDM) samples of VRZ from these participants. A one-compartment model with first-order absorption and elimination described the population pharmacokinetics of VRZ. Population estimates for apparent clearance (CL/F) and volume of distribution (V/F) were 17.9 L/h/70kg (RSE, 10.8%) and 788 L/70kg (RSE, 15.4%), respectively. An XGBoost model accurately predicted voriconazole concentrations (R^2^ = 0.81, RMSE = 0.53) with a relative error of ±20% for most observations. In the external validation, the XGBoost model demonstrated an R^2^ of 0.75, RMSE of 0.14. SHAP analysis identified clearance, weight, and laboratory values as significant predictors.

**Conclusion:**

This study emphasized the importance of personalized treatment in utilizing VRZ for children under 24 months. The XGBoost model demonstrated potential in identifying an initial dose recommendation for VRZ.

## 1 Introduction

Voriconazole (VRZ), a triazole antifungal, is employed extensively in the management of invasive fungal infections among pediatric patients (Kadam and Van Den Anker, 2016), particularly those with weakened immune systems due to conditions such as hematologic malignancies or after undergoing hematopoietic stem cell transplantation (HSCT). Although approved by the US FDA and European Medicines Agency (EMA) for children aged two and older, a scarcity of comprehensive pharmacokinetic data complicates its use in infants under two (Chen et al., 2018). This data gap requires that clinicians carefully weigh the potential therapeutic benefits for an individual patient against the known pharmacokinetic variability, leading them to frequently resort to its “off-label” use after a careful risk-benefit assessment. In such cases, therapeutic drug monitoring (TDM) is essential for addressing the high pharmacokinetic variability in this young population and for optimizing both safety and efficacy (Touw and van den Anker, 2022). Pediatric patients, especially those below 24 months, exhibit different pharmacokinetic profiles compared to older children and adults, necessitating careful consideration of dosing strategies (Yan et al., 2018; Gastine et al., 2023). Although effective, VRZ’s pharmacokinetics (PK) in children showed considerable variability, frequently influenced by age, body weight, and genetic polymorphisms (Soler-Palacin et al., 2012). This challenge is magnified in children under 2 years old, where profound PK variability - driven by developmental changes and genetic polymorphisms - makes optimal dosing difficult.

The pharmacokinetics of VRZ may vary significantly among patients, especially in critically ill individuals, pediatrics, the elderly, and the obese (Wang et al., 2025; Jiang et al., 2022). Understanding VRZ population pharmacokinetics is essential for optimal dosing, given its narrow therapeutic index and patient variability from factors like C-reactive protein levels and genetic polymorphisms (van den Born et al., 2023; Li et al., 2023). Population pharmacokinetic analysis is a powerful approach that effectively addresses dosing challenges by understanding voriconazole behavior and providing insights for optimizing clinical dosing strategies, even with limited sampling data. However, classical PopPK methods rely on fixed compartmental models that may limit the ability to fully capture all factors influencing drug behavior, particularly when working with sparse sampling data (Liu et al., 2025).

ML, a core branch of artificial intelligence, is adept at capturing complex patterns and nonlinear relationships within big data (Asnicar et al., 2024; Greener et al., 2022). ML techniques are emerging as valuable complements to PopPK approaches in optimizing voriconazole dosing. For instance, a recent study (Cheng et al., 2023) demonstrated that machine learning algorithms, particularly XGBoost, could predict toxic plasma trough concentrations of VRZ (>5 mg/L) with an accuracy of 78.8%, identifying key factors such as albumin and total bile acid for dosage optimization. Utilizing pharmacokinetic (PK) parameters in machine learning models is an innovative strategy that has been proven effective with various drugs (Li et al., 2024; Ma et al., 2024; Huang et al., 2022), leading to improved model predictive accuracy and enhanced model interpretability.

**FIGURE 1:**
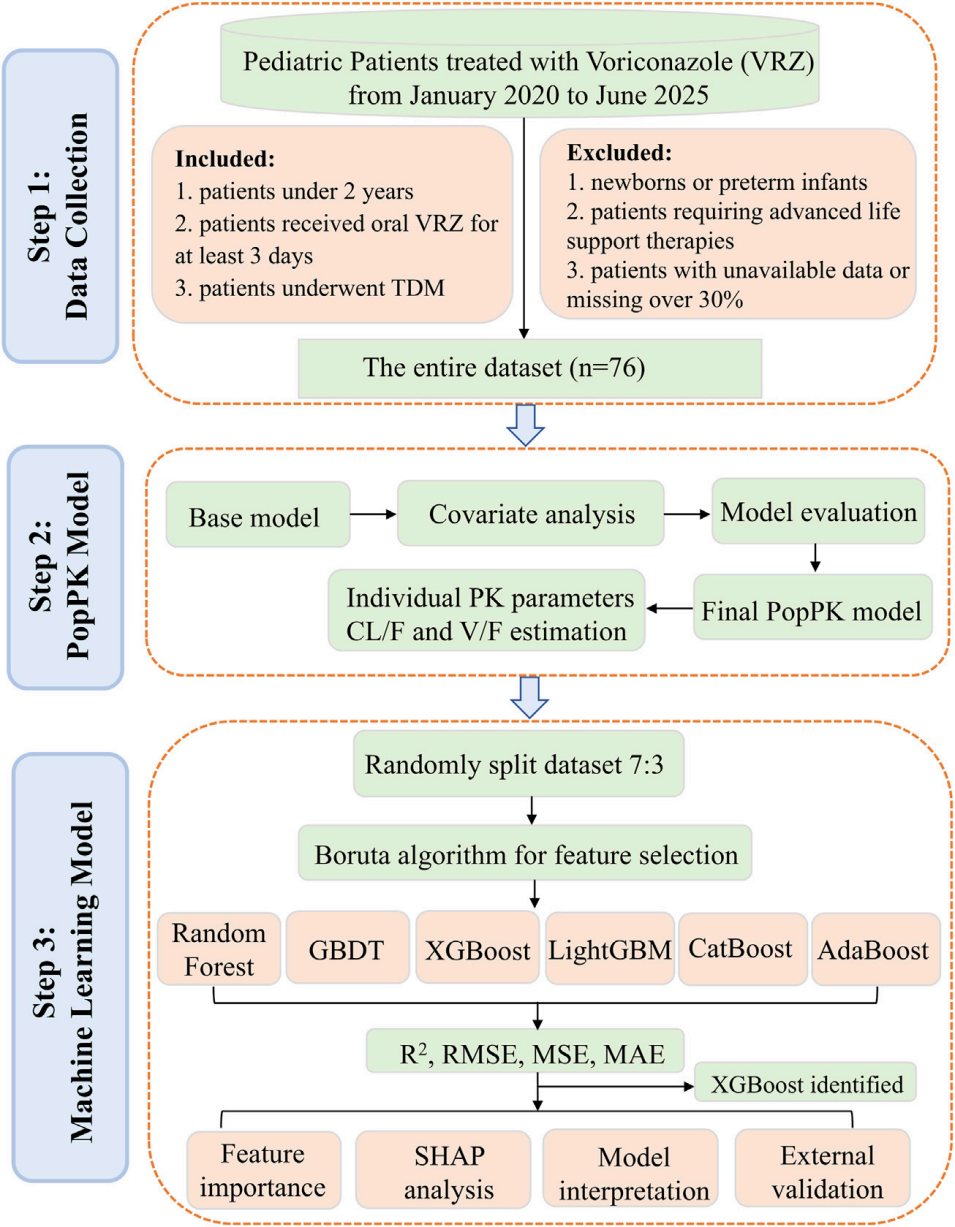
Study process workflow.

As VRZ is a crucial option for managing invasive fungal diseases, and considering the limited data on dosing and exposure in children under 24 months, this study aims to share experiences with individualized voriconazole therapy. We developed a model that integrate PopPK with ML approaches to predict the steady-state plasma concentration of oral VRZ in this age group. The study workflow was presented in Figure 1.

## 2 Methods

### 2.1 Study design

We conducted a retrospective observational study analyzing data from pediatric patients aged under 24 months who had been hospitalized at the Children’s Hospital of Fudan University from January 2020 to June 2025. Inclusion criteria included patients aged below 2 years who received oral VRZ for a minimum of 3 days during their hospitalization and underwent therapeutic drug monitoring (TDM) (Chen et al., 2018; Resztak et al., 2021). Exclusion criteria included newborns, preterm infants, patients requiring advanced life support therapies, and patients whose medical information was unavailable or missing more than 30%. For independent external validation, we also collected data from 10 patients under 24 months who received oral VRZ and underwent TDM at our hospital between July and August 2025.The ethics committee of Children’s Hospital of Fudan University approved the study protocol [No. (2024) 321]. Informed consent was waived for the collection and analysis of the anonymous data, as there was no intervention involved, and the study posed minimal risk (Filion et al., 2016; Strutz et al., 2025).

For patients who satisfied the enrollment criteria, a comprehensive set of data was collected from their electronic medical records. Clinical characteristics included age, sex, height (HT), and weight (WT), from which the body surface area (BSA) was calculated using the formula: BSA (m^2^) = $\sqrt{\frac{\text{HT}\left(\text{cm}\right)\times \text{WT}\left(\text{kg}\right)}{3600}}$ . Additionally, a panel of laboratory indices was recorded, including white blood cell count (WBC), neutrophil percentage (N%), lymphocyte percentage (L%), red blood cell count (RBC), hemoglobin (HGB), platelet count (PLT), C-reactive protein (CRP), albumin (ALB), total bilirubin (TBIL), direct bilirubin (DBIL), alanine aminotransferase (ALT), aspartate aminotransferase (AST), alkaline phosphatase (ALP), serum creatinine (SCR), international normalized ratio (INR), and D-Dimer. Each TDM sample represented an independent sampling event. Laboratory values were collected on the same day as or within 24 h of the TDM sample. Furthermore, treatment-related data for VRZ were documented, comprising the total daily dose, dosing interval, therapy duration at TDM, the timing of the last dose before blood sampling, the measured trough concentration (mg/L), and all co-administered therapies. The estimated glomerular filtration rate (EGFR), with k = 0.45 for children under 1 year and k = 0.413 for children aged 1–2 years, was determined using the Schwartz formula (Schwartz et al., 1984; Schwartz et al., 2009).

Treating physicians determined VRZ doses individually for each patient, considering clinical status and therapeutic drug monitoring. While referencing the standard 9 mg/kg q12h regimen for older children (2 to <12 years), oral doses for children under 2 years ranged from 4 to 10 mg/kg every 12 h. This dosing strategy is consistent with recommendations from UpToDate, a clinical decision support system (Isaac et al., 2012). Blood samples were collected to determine steady-state trough concentrations, with sampling performed within the 30-min window immediately preceding the next scheduled dose. In cases of missing covariate data, which accounted for less than 3% of the dataset, median imputation was employed (Shen et al., 2024).

### 2.2 VRZ therapeutic drug monitoring

The plasma concentration of VRZ was determined by high-performance liquid chromatography (HPLC) using a Shimadzu LC-20 series HPLC system (Shimadzu, Japan). A Waters XBridge C18 chromatographic column (4.6 × 150mm, 5 μm) was used. The mobile phase A consisted of water, while mobile phase B was acetonitrile. The injection volume was 20 μL, with the detection wavelength fixed at 256 nm. The standard curve for VRZ covered a linear range of 0.25–10 mg/L. The target concentration for VRZ was set between one and 5.5 mg/L in plasma trough (Pappas et al., 2016).

### 2.3 Population pharmacokinetic modeling

The PopPK analysis of VRZ was carried out in NONMEM^®^ software (version 7.5.0, ICON plc., USA) executing the first-order conditional estimation method with interaction (FOCE-I). The pharmacokinetic profile of VRZ was characterized using the ADVAN2 TRANS2 subroutine to develop the basic structural model, representing a one-compartment disposition with first-order absorption and elimination. The pharmacokinetic parameters assessed were the apparent clearance (CL/F), the apparent volume of distribution (V/F), and the absorption rate constant (Ka). Due to the sampling design primarily focusing on steady-state trough concentrations, individualized absorption parameters could not be reliably assessed. Ka was fixed to 1.19 h^-1^, reflecting published literature values (Gastine et al., 2018). An exponential model was used to quantify interindividual variability in CL/F and V/F. Various residual error models (proportional, additive or combined) were evaluated to assess intra-individual variability. Using allometric scaling, we examined the effects of body weight on PK parameters.

Covariates were evaluated utilizing a stepwise forward-addition/backward-elimination approach based on objective function value (OFV) changes. During forward selection, covariates were included if they reduced the OFV by > 3.84 (p < 0.05). In the backward elimination phase, covariates were retained in the final model only if their removal increased the OFV by > 6.63 (p < 0.01). Final model evaluation was conducted through diagnostic goodness-of-fit (GOF) plots, bootstrap analysis, and visual predictive check (VPC). Parameter precision was evaluated, and 95% confidence intervals (95%CI) were generated using bootstrap resampling (n = 1000). VPC analysis was performed using 1000 simulated datasets to characterize the distribution characteristics of observed versus simulated data. The final PopPK model enabled the calculation of pediatric individual PK parameters CL and V via the empirical Bayesian estimates method. These PK parameters were subsequently introduced as features in the ML model.

### 2.4 Machine learning modeling

For model development and validation, the dataset was randomly partitioned into a training set (70%) and a validation set (30%). The Boruta algorithm was employed for feature selection to identify all relevant features (Shen et al., 2024). The Boruta algorithm automates feature selection by evaluating the significance of original features against shadow features (Li et al., 2025). The Boruta was executed for 200 iterations with a significance threshold of p < 0.01, applying Bonferroni correction for multiple comparisons to identify statistically significant features. Six ML algorithms were chosen to construct models: extreme gradient boosting (XGBoost), light gradient boosting machine (LightGBM), gradient boosting decision tree (GBDT), categorical boosting (CatBoost), adaptive boosting (AdaBoost), and random forest (RF). To evaluate the stability and generalizability of our final model, we performed a 10-iteration bootstrap cross-validation and an independent external validation.

Model performance was assessed using four standard regression metrics: Mean Squared Error (MSE), Root Mean Squared Error (RMSE), Mean Absolute Error (MAE), and coefficient of determination (R^2^), providing a comprehensive evaluation of predictive accuracy. Lower MSE, RMSE, and MAE values signify enhanced model performance, whereas R^2^ values approaching one indicate greater explanatory power. After establishing the optimal model, we used relative accuracy (RA) as an indicator to evaluate the model’s predictive performance (Huang et al., 2020). In this study, RA was measured by whether the relative error between VRZ predicted values and observed values falls within a specified percentage threshold of 20%. The optimal model was chosen for interpretability analysis using the Shapley Additive Explanations (SHAP) method. The approach assessed each feature’s impact on specific predictions, offering insights into both local and global model behavior (Qi et al., 2025).

### 2.5 Statistical analysis

Categorical variables were presented using frequencies and percentages [N (%)]. Continuous variables were described using both medians with interquartile ranges [Median (IQR)] and means with standard deviations [Mean ± SD]. The Kruskal–Wallis test was used to analyze continuous variables, while Fisher’s exact test was employed for categorical variables. A p-value of below 0.05 was established as the threshold for statistical significance. VRZ plasma concentrations below the lower limit of quantitation (LLOQ) of 0.25 mg/L were labeled as below quantitation limit (BQL). These BQL values were substituted with half the LLOQ value (0.125 mg/L) (Chabala et al., 2022). In this study, BQL data accounted for less than 4% of the total observations. R software (v 4.5.0) and Python (v 3.10.6) were utilized for all analyses.

## 3 Results

### 3.1 Clinical characteristics of pediatric patients

The clinical and demographic characteristics of the 76 pediatric patients, from whom 110 samples were collected, were summarized in Table 1. The study population consisted of 58 males (76.3%) and 18 females (23.7%). The median age, height, and weight were 11.0 months (IQR, 7.38–17.00), 70.00 cm (IQR, 66.00–74.00), and 8.05 kg (IQR, 6.95–9.00), respectively. The median voriconazole plasma concentration was 1.25 mg/L (IQR, 0.66–2.58). At the time of sampling, patients were receiving a median total daily VRZ dose of 100.00 mg (IQR, 100.00–133.25) for a median duration of 9.50 days (IQR, 5.00–17.75). Crucial laboratory values included a median WBC of 8.04 × 10^9^/L (IQR, 4.71–12.38) with a median neutrophil percentage of 52.58% (IQR, 34.80–66.62), and a median HGB level of 105.50 g/L (IQR, 94.92–116.25). The median ALT and SCR were 39.50 U/L (IQR, 20.31–81.80) and 18.92 μmol/L (IQR, 15.40–22.54), respectively. The most common co-administered drugs were glucocorticoids (47 patients, 61.8%), proton pump inhibitors (34, 44.7%) and tacrolimus (25, 32.9%). For model development, the dataset was randomly partitioned into a training set and a validation set at a 7:3 ratio, with comparable characteristics detailed between the two sets in Supplementary Table S1. We assessed hepatic function parameters (ALT, AST, ALP) and found no significant changes between baseline and maximum values during voriconazole therapy (Supplementary Figure S1). Additionally, breakthrough fungal infections were not observed.

**TABLE 1:** Clinical characteristics of the study population.

Characteristic	Mean (SD)	Median	IQR
No. of patients/samplings	76/110		
Gender (Boys/Girls)	58/18		
Age, months	12.08 (5.94)	11.00	7.38–17.00
Height, cm	69.97 (6.84)	70.00	66.00–74.00
Weight, kg	8.00 (1.91)	8.05	6.95–9.00
BSA, m^2^	0.39 (0.07)	0.39	0.36–0.43
VRZ concentration, mg/L	1.63 (1.41)	1.25	0.66–2.58
Therapy duration at TDM, days	12.73 (9.97)	9.50	5.00–17.75
Total daily dose, mg	112.59 (30.11)	100.00	100.00–133.25
WBC, 10^9^/L	9.16 (5.71)	8.04	4.71–12.38
N%	51.64 (20.11)	52.58	34.80–66.62
L%	33.68 (20.59)	32.69	15.70–49.75
RBC, 10^12^/L	3.84 (0.78)	3.83	3.35–4.28
HGB, g/L	106.02 (16.46)	105.50	94.92–116.25
ALB, g/L	36.94 (4.13)	37.48	34.58–39.62
PLT, 10^9^/L	293.49 (205.11)	304.5	77.37–470.50
CRP, mg/L	11.13 (26.13)	1.74	0.50–6.92
TBIL, μmol/L	11.65 (21.46)	5.35	3.18–11.01
DBIL, μmol/L	7.61 (17.86)	2.40	1.40–5.66
ALT, U/L	68.98 (85.55)	39.50	20.31–81.80
AST, U/L	89.96 (100.93)	56.14	37.79–110.92
ALP, U/L	222.50 (123.12)	211.26	121.38–263.75
SCR, μmol/L	19.20 (5.34)	18.92	15.40–22.54
EGFR, mL/min/1.73 m^2^	151.92 (51.25)	139.18	115.39–169.90
INR	1.09 (0.71)	0.97	0.91–1.04
D-Dimer, mg/L	1.00 (1.43)	0.64	0.45–0.92
Co-medication (n, %)			
Tacrolimus	25 (32.89%)		
Sirolimus	2 (2.63%)		
Cyclosporine A	10 (13.33%)		
Proton pump inhibitor	34 (44.74%)		
Glucocorticoids	47 (61.84%)		

Abbreviations: SD, standard deviation; IQR, interquartile range; TDM, therapeutic drug monitoring; BSA, body surface area; WBC, white blood cell count; N%, neutrophil percentage; L%, lymphocyte percentage; RBC, red blood cell count; HGB, hemoglobin; ALB, albumin; PLT, platelet count; CRP, C-reactive protein; TBIL, total bilirubin; DBIL, direct bilirubin; ALT, alanine aminotransferase; AST, aspartate aminotransferase; ALP, alkaline phosphatase; SCR, serum creatinine; INR, international normalized ratio; EGFR, estimated glomerular filtration rate.

### 3.2 PopPK analysis

In the final PopPK model assessed the inter-individual variability (IIV) for CL/F through the variance component ω^2^_CL/F_, estimated at 0.674, indicating a standard deviation of approximately 0.821, with an RSE% of 10.7%. However, IIV was not estimated for V/F due to high shrinkage (>90%) and an RSE exceeding 30%. The final model yielded a population typical value of CL/F at 17.9 (L/h/70kg) and a V/F of 788 (L/70kg), with RSE% values of 10.8% and 15.4%, respectively, as detailed in Supplementary Table S3 and Equations 1, 2. The GOF plots and VPC for our final model were illustrated in Supplementary Figures S2, S3. Bootstrapping validation (100% minimization success) of the population typical value estimates for CL/F indicated a median of 17.65 (L/h/70kg) with a 95% CI of 9.11–22.00 (L/h/70kg). Meanwhile, the median for V/F was 787.75 (L/70kg), with a 95% CI of 94.82–1050.85. The final PopPK model was as follows:

$$\text{CL}/F\;\left(L/h/70\mathrm{kg}\right)=17.9\times {\left(\text{WT}/70\right)}^{0.75}$$ (1)

$$V/F\;\left(L/70\mathrm{kg}\right)=788\times \left(\text{WT}/70\right)$$ (2)

### 3.3 Feature selection and model construction

**FIGURE 2:**
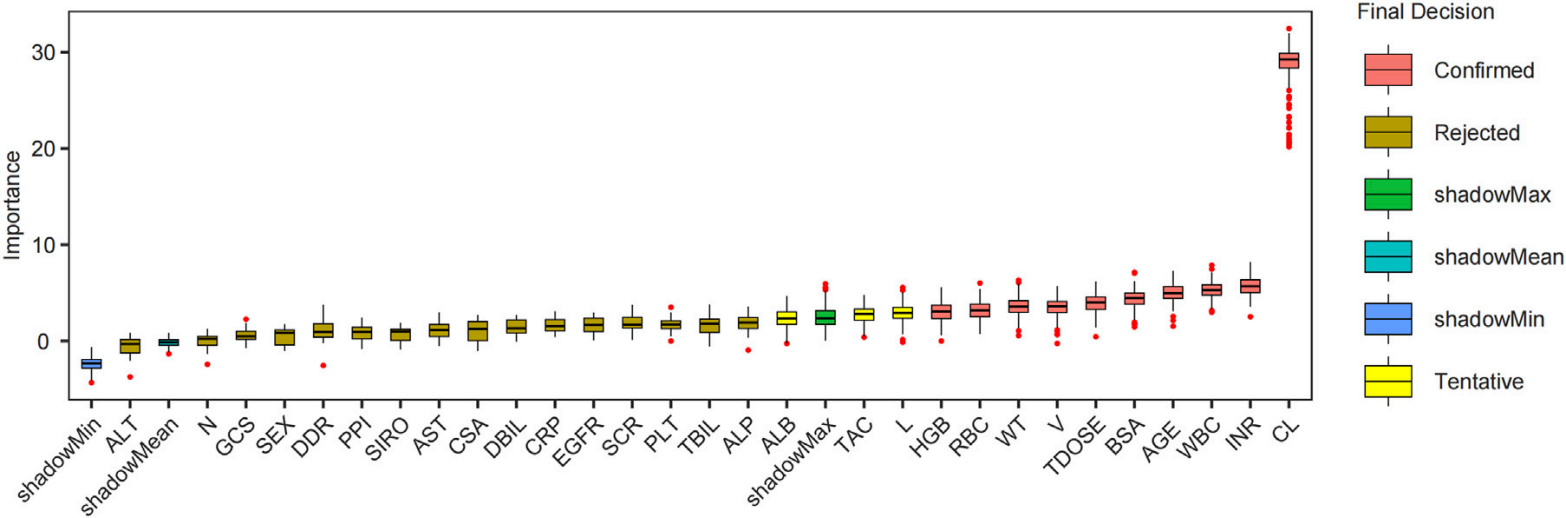
Boruta feature filtering each variable importance box plot. Abbreviations: CL, clearance; INR, international normalized ratio; WBC, white blood cell count; BSA, body surface area; TDOSE, Total daily dose; V, volume of distribution; WT, weight; RBC, red blood cell count; HGB, hemoglobin; L, lymphocyte percentage; TAC, tacrolimus; ALB, albumin; ALP, alkaline phosphatase; TBIL, total bilirubin; PLT, platelet count; SCR, serum creatinine; CSA, Cyclosporine A; AST, aspartate aminotransferase; SIRO, sirolimus; PPI, proton pump inhibitor; DDR, D-dimer; GCS, glucocorticoids; N, neutrophil percentage; CRP, C-reactive protein; DBIL, direct bilirubin; ALT, alanine aminotransferase; EGFR, estimated glomerular filtration rate.

The Boruta algorithm, employed for covariate screening across 200 iterations, identified 10 clinically significant variables that outperformed the shadow attributes (Figure 2). These variables included PK parameters such as CL and V, hematologic indices (INR, WBC, RBC, and HGB), as well as age, BSA, weight, and total daily dose. These ten variables were subsequently selected for the further development of the ML model.

**FIGURE 3:**
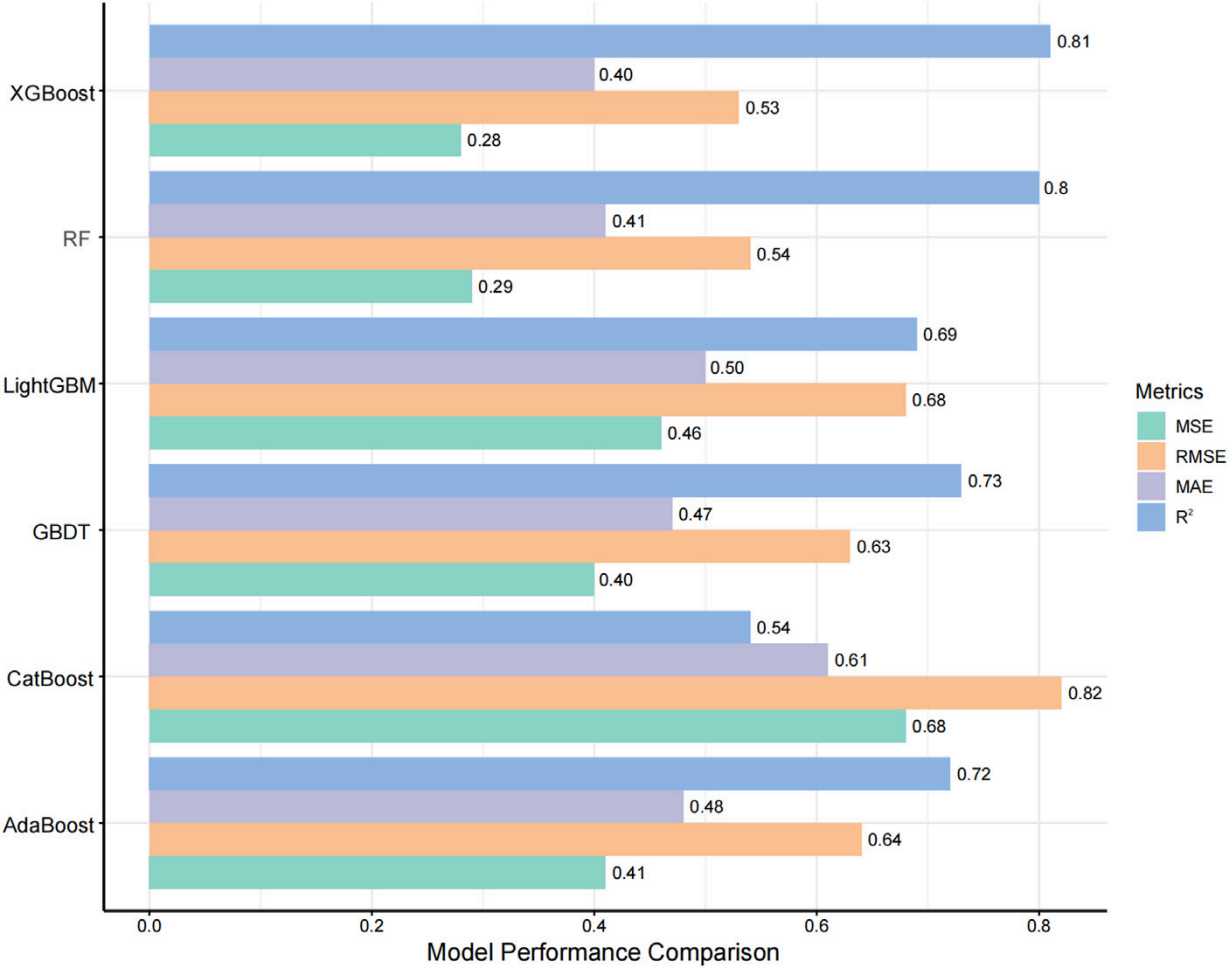
Comparison of Model Performance Metrics. The ranges for MSE, RMSE, MAE, and R^2^ are all between 0 and 1, where a higher R^2^ indicates better model performance, and lower values of MSE, RMSE, and MAE indicate better accuracy. Abbreviations: GBDT, gradient boosting decision tree; RF, random forest. MSE, mean squared error; RMSE, root mean squared error; MAE, mean absolute error.

**FIGURE 4:**
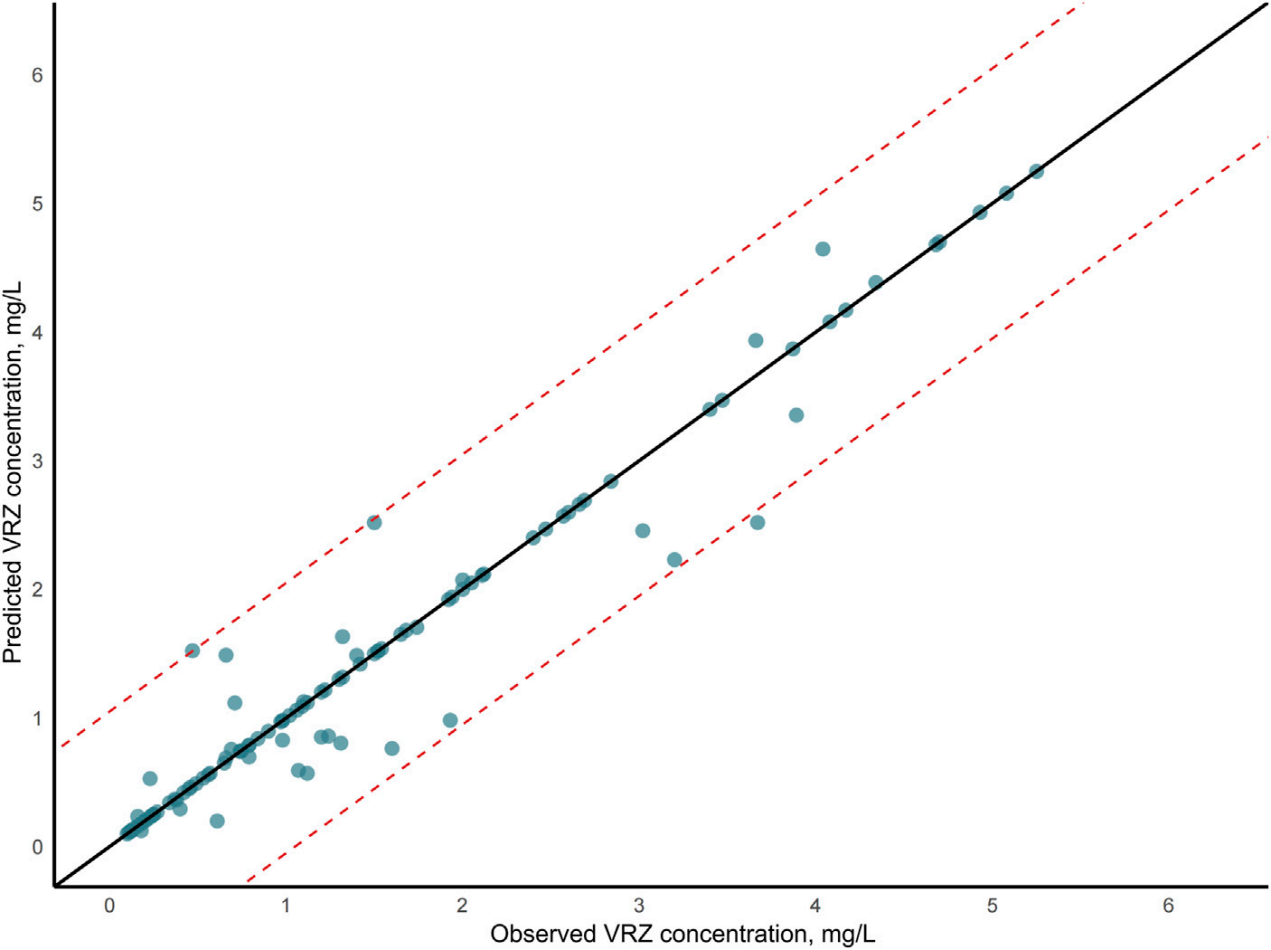
Voriconazole (VRZ) plasma concentration observations versus predicted concentration based on the XGBoost algorithm. In the scatter plot, the two dashed lines represented the ±20% relative error thresholds.

The evaluation of model performance was shown in Figure 3 and Supplementary Table S4. Among the six algorithms assessed, XGBoost emerged as the optimal model, with an R^2^ value of 0.81, MSE of 0.28, RMSE of 0.53, and MAE of 0.40. Random Forest (RF) demonstrated comparable accuracy with an MSE of 0.29 and an R^2^ of 0.80, indicating a strong predictive capability. The robustness of the final model was confirmed via a 10-iteration bootstrap cross-validation, which yielded a stable mean R^2^ of 0.805 (range: 0.776–0.867), as detailed in Supplementary Table S5. The characteristics of the external validation population (n = 10) were summarized in Supplementary Table S2, including a median age of 14 months (IQR, 5.50–21.50) and a median weight of 7.75 kg (IQR, 6.00–10.23). Furthermore, on the external validation dataset, the XGBoost model achieved an R^2^ of 0.75, RMSE of 0.14, MSE of 0.02, and MAE of 0.12. The VRZ plasma concentration observations were contrasted with predictions based on the XGBoost algorithm, as shown in Figure 4. The majority of data points fall within the indicated ±20% relative error thresholds. Considering the model’s prediction accuracy and goodness of fit, the XGBoost was selected for further interpretability analysis.

### 3.4 Model interpretation

**FIGURE 5:**
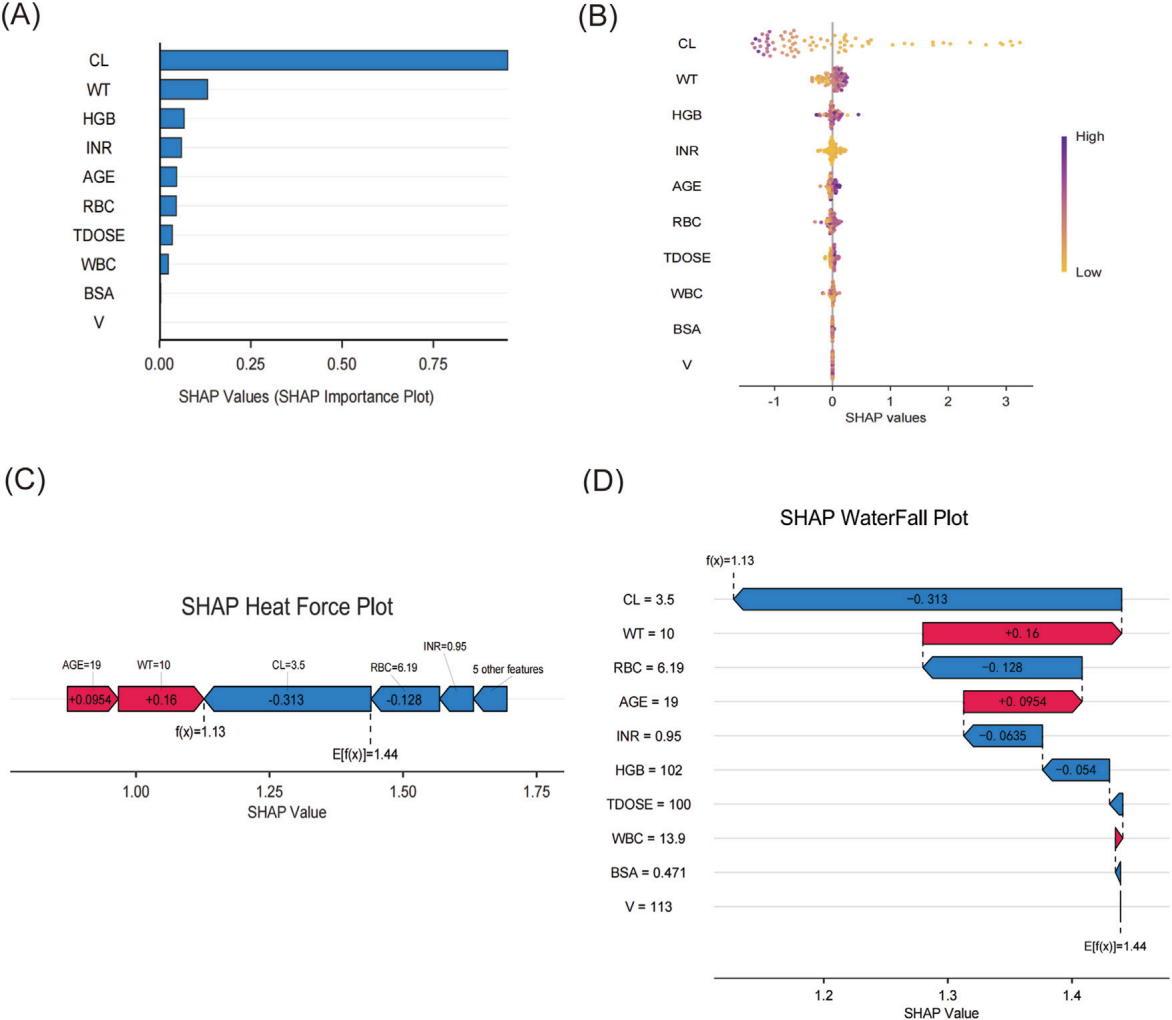
The SHAP analysis of the XGBoost. **(A)** Bar plot of feature importance in XGBoost model predictions based on SHAP Values. **(B)** The beeswarm plot showed the SHAP values for ten features in the XGBoost model. The darker the color, the more important the variable was. **(C)** The SHAP heat force plot. **(D)** The SHAP waterfall plot.

**FIGURE 6:**
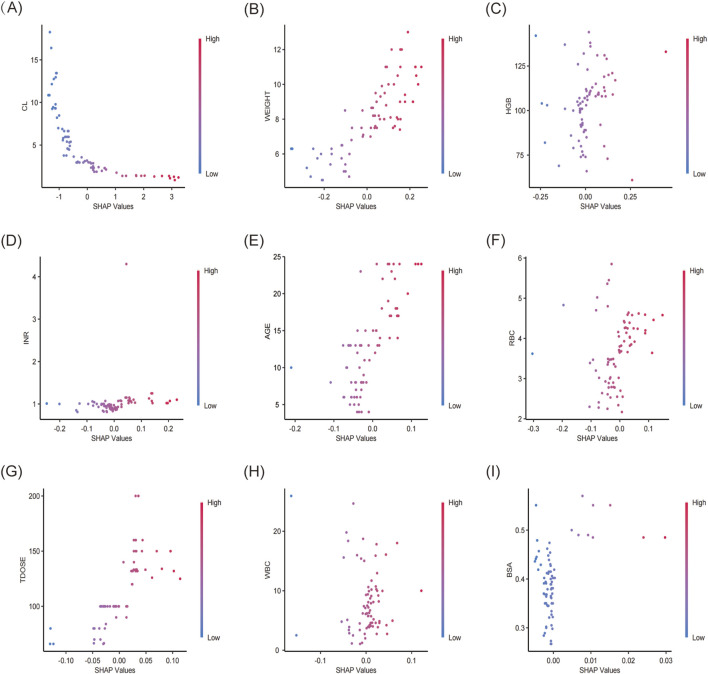
SHAP dependence plot of the XGBoost model. The x-axis showed the SHAP values of each variable, while the y-axis displayed the raw values. SHAP dependence plots for **(A)** CL, clearance, **(B)** weight, **(C)** HGB, hemoglobin, **(D)** INR, **(E)** age, **(F)** RBC, red blood cell count (RBC), **(G)** TDOSE, total daily dose, **(H)** WBC, white blood cell count, **(I)** BSA, body surface area.

The XGBoost model was employed to predict plasma concentrations of oral VRZ in children under 2 years old, alongside an analysis of model interpretability. Global feature contributions, as determined by SHAP analysis, were presented in Figure 5. Clearance (CL) was identified as the most important feature, followed by weight (WT), hemoglobin (HGB), international normalized ratio (INR), and age, based on mean absolute SHAP values (Figure 5A). The beeswarm plot (Figure 5B) visualized the magnitude and direction of these contributions for each individual, confirming the substantial and heterogeneous impact of CL and WT, as evidenced by their wide distribution of SHAP values. Figure 6 displayed the SHAP dependence plots, illustrating the relationship between feature values and their influence on predictions made by the XGBoost model. The findings demonstrated a negative correlation, suggesting that higher CL values were associated with lower VRZ concentrations.

Figure 5C displayed a SHAP waterfall plot, which deconstructed the XGBoost model’s prediction for a pediatric patient (age 19 months, weight 10 kg) receiving oral VRZ. This visualization illustrated the stepwise derivation of the patient’s individualized concentration prediction (f(x) = 1.13 mg/L) from the population mean baseline (E [f(x)] = 1.44 mg/L). The analysis identified VRZ clearance (CL = 3.5 L/h/70kg) as the predominant driver of the prediction, contributing a SHAP value of −0.313. Conversely, the patient weight = 10 kg exerted a positive influence (SHAP = +0.16). Other covariates with negative contributions included RBC = 6.19 × 10^9^/L (SHAP = −0.128), INR = 0.95(SHAP = −0.0635), and HGB = 102 g/L (SHAP = −0.054). Patient age (19 months) had a modest positive impact (SHAP = +0.0954). As a complementary visualization, the SHAP force plot (Figure 5D) provides a consolidated view, illustrating how these positive (red) and negative (blue) forces balance to produce the final output.

### 3.5 Clinical application

**FIGURE 7:**
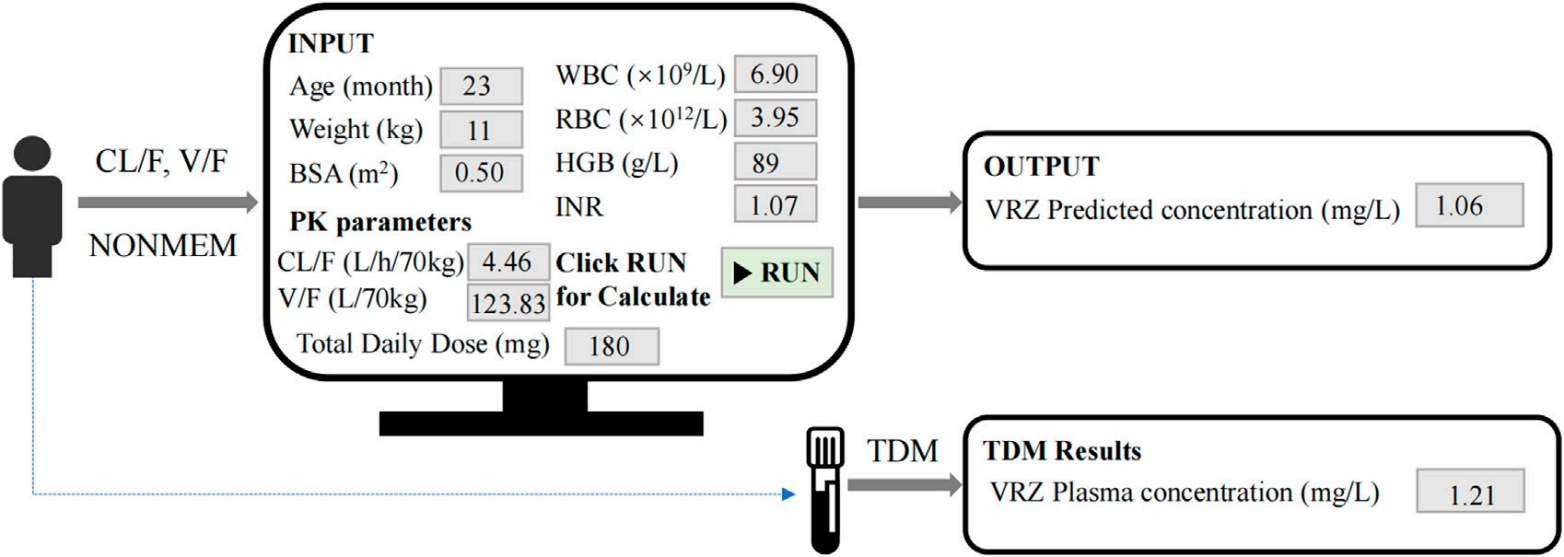
Clinical application of the model.

Figure 7 illustrated the process of predicting VRZ plasma concentration using our model. For instance, the model utilized patient-related parameters, including age (23 months), weight (11 kg), BSA (0.5 m^2^), WBC (6.9 × 10^9^/L), RBC (3.95 × 10^12^/L), HGB (89 g/L), INR (1.07), CL/F (4.46 L/h/70kg), V/F (123.83 L/70kg), and total daily dose (180 mg). Given the initial dose of 180 mg daily, administered as 90 mg twice daily, this corresponds to a minimum dosage of 8 mg/kg (8.18 mg/kg). By inputting these parameters and running the model, the VRZ concentration was predicted to be 1.06 mg/L. Three days later, TDM results indicated an actual VRZ plasma concentration of 1.21 mg/L. This model is expected to assist clinicians in making initial VRZ dosage recommendations to rapidly achieve the therapeutic concentration window before TDM results are available.

## 4 Discussion

VRZ is a critical therapeutic agent for invasive fungal infections; however, evidence-based dosing guidelines and drug exposure data in children younger than 24 months are scarce. To address this knowledge gap, we developed a model integrating PopPK with ML to estimate steady-state plasma concentrations of oral VRZ in this vulnerable pediatric population. This study enrolled 76 pediatric patients, including 58 males (76.32%) and 18 females (23.68%), with a median age of 11 months and a weight of 8.1 kg. Using a Bayesian PopPK approach, we estimated individual clearance and volume of distribution, incorporating these PK parameters as features in our ML model. Prior to model development, the Boruta algorithm was used for feature selection, resulting in 10 variables being included in the final ML model. The XGBoost algorithm demonstrated optimal predictive ability for VRZ plasma concentrations in children under 2 years, with an R^2^ of 0.81 and an MSE of 0.28. SHAP analysis identified clearance, weight, and HGB as the top three significant predictors of VRZ concentrations.

Previous studies have investigated VRZ dosing and plasma concentrations in diverse patient populations. For instance, Gastine *et al.* examined VRZ plasma concentrations in 17 children under 24 months across 50 distinct treatment episodes, reporting a median trough concentration of 0.63 mg/L, with only 34.2% of samples reaching the recommended therapeutic range (Gastine et al., 2023). In contrast, our study involved a larger population of 76 pediatric patients, potentially enhancing the reliability and general applicability of our findings. Huang *et al.* assessed the efficacy of ML models versus traditional PopPK models for predicting VRZ trough concentrations in critically ill patients (Huang et al., 2025). Utilizing a dataset of 244 concentrations from 62 patients, they developed 6 ML models, with the XGBoost model demonstrating superior predictive performance (R^2^ = 0.73). Similary, Liu *et al.* developed a real-time ML ensemble model to forecast VCZ plasma concentrations in elderly patients, achieving an R^2^ of 0.828 by reducing features from 31 to nine while maintaining predictive accuracy (Liu et al., 2025). In our analysis, the XGBoost model achieved an R^2^ of 0.81 and an MSE of 0.28, indicating comparable predictive capability. Furthermore, our SHAP analysis evaluated the influence of several variables, including clearance, body weight, and hemoglobin levels, on VCZ concentrations, providing clinical interpretations of feature importance.

In our PopPK model, body weight was incorporated as a covariate influencing VRZ clearance. Numerous factors have been reported to affect VRZ metabolism, contributing to variability in blood concentrations. Patient demographics significantly influence voriconazole pharmacokinetics. Age-related differences are notable, with pediatric patients requiring higher weight-based dosing due to faster clearance compared to adults (Tilen et al., 2022). The PopPK study by Wang *et al.* identified clearance as a pivotal parameter, significantly correlating with age, ALP levels, and CYP2C19 genotype (Wang et al., 2014). Johnson *et al.* found that CL was significantly negatively correlated with INR, total bilirubin, and AST in liver transplant patients (P < 0.05), suggesting that liver function indicators may indirectly regulate drug concentration by affecting clearance (Johnson et al., 2010). Consistent with these findings, our final PopPK model estimated a population typical value of clearance (CL/F) at 17.9 L/h/70kg (RSE, 10.8%). Bootstrapping validation indicated a median CL/F of 17.65 L/h/70kg (95% CI, 9.11–22.00 L/h/70kg). These results further underscore the importance of clearance as a key factor of VRZ concentrations, particularly in the pediatric population.

SHAP analysis identified CL as the most significant factor influencing VRZ concentration, consistent with the substantial interindividual variability observed in clearance among individuals. This variability likely reflects underlying physiological differences such as liver function, which is primarily responsible for metabolizing voriconazole (Hashemiza et al., 2017), as well as interindividual differences in CYP450 enzyme activity. Studies have confirmed that liver dysfunction can complicate clearance metrics, necessitating adjustments to dosing to avoid subtherapeutic levels and toxicity (Gorski et al., 2011; Wang et al., 2018). While CL was the predominant factor, other features also contributed to VRZ concentration variability. Body weight (WT) plays a significant role in individualizing VRZ dosing regimens, particularly in pediatric populations. A clinical study observed that obese individuals demonstrated reduced VRZ metabolism compared to their non-obese counterparts, which was associated with higher VRZ trough concentrations (Takahashi et al., 2020). Conversely, Patients with lower body weight may experience insufficient drug exposure, leading to lower plasma drug concentrations (Tilen et al., 2022), particularly in those with a body surface area under 1 m^2^. Children metabolize VRZ at a faster rate due to higher metabolic activity and different body compositions compared to adults, necessitating more intensive dosing strategies to reach target plasma concentrations effectively (Neely et al., 2010; Shima et al., 2010). Hemoglobin (HGB) levels can influence pharmacokinetics indirectly through their association with blood volume and systemic inflammatory responses. Although the effect size of HGB was smaller than that of CL in our study, studies have suggested associations between HGB levels and altered pharmacokinetics, particularly in populations at risk for anemia (Hsu et al., 2015). Anemia or elevated WBC and RBC counts might reflect systemic inflammation or altered drug distribution, affecting VRZ concentrations (Neely et al., 2010). Age also significantly affects VRZ pharmacokinetics, with pediatric patients requiring different dosing strategies due to metabolic differences compared to adults. As patients age, VRZ clearance can change, necessitating tailored dosing to maintain efficacy without increasing toxicity (Chen et al., 2022; Bartelink et al., 2013). While CL remains the dominant factor, VRZ concentration variability, as revealed by our SHAP analysis, is meaningfully influenced by a combination of body weight, hemoglobin levels, age, and inflammatory markers; thus, these factors warrant consideration in individualized dosing, especially for vulnerable populations.

The study had several limitations. First, as a single-center, retrospective analysis, the findings may have limited generalizability. Furthermore, the study population comprised exclusively of Asian children; PK differences across ethnic groups suggest that the model’s applicability to non-Asian pediatric populations requires further investigation (Chen et al., 2018; Weiss et al., 2009). Additionally, Voriconazole plasma concentrations were measured using an assay established within our laboratory. Variations in reagents, instrumentation, and potential matrix effects between laboratories may lead to systematic differences in model performance at other centers.

The trough-only sampling design resulted in a wide 95% confidence interval for the apparent volume of distribution (V/F 94.82–1050.85L/70kg). While this approach optimized the estimation of voriconazole clearance (CL/F), it provided limited information regarding drug distribution. Nevertheless, the stability of the overall model structure and the reliability of key parameter estimates were supported by a 100% success rate in bootstrap validation. Moreover, our findings were consistent with previously reported high inter-individual variability and allometric scaling in pediatric populations (Friberg et al., 2012).

Additionally, the model did not include genotype features related to VRZ metabolism, such as CYP2C19, which may limit a comprehensive understanding of drug metabolism (Moriyama et al., 2017; Caudle et al., 2025). However, it is important to emphasize that CYP2C19 genotyping has not yet been established as a standard clinical practice in most healthcare settings, presenting a challenge for the widespread adoption of genotype-guided VRZ dosing strategies. Therefore, the primary objective of this study was to develop a clinically applicable model using readily available clinical data to predict VRZ concentrations in children under 2 years of age. This model is intended to help clinicians rapidly achieve therapeutic VRZ concentrations by informing initial dosage recommendations before therapeutic drug monitoring (TDM) results are available. As a National Children’s Medical Center, we are leading a multicenter, prospective study to systematically evaluate the efficacy and safety of VRZ in young children. Ultimately, we aim to develop a user-friendly web application to facilitate the model’s implementation in clinical practice.

## 5 Conclusion

This study represented the first effort to merge classic PopPK with ML to develop a predictive model for oral VRZ plasma concentrations in children under 24 months. A one-compartment model of voriconazole was established for this age group, revealing that weight significantly impacts CL/F and V/F. Individual CL and V were estimated using the empirical Bayesian method, which were then incorporated into the ML modeling process.

We used the Boruta algorithm for feature selection and constructed prediction models for VRZ plasma concentrations employing various ML algorithms. Among these, XGBoost demonstrated the best performance, achieving an R^2^ value of 0.81 and a mean squared error of 0.28. SHAP explainability analysis indicated that clearance, weight, hemoglobin, and other factors were most influential in the model.

Our findings provide a significant reference for individualized voriconazole dosing in clinical practice for children under 2 years old. A potential benefit of the model lies in its capacity to suggest an initial dose before TDM results are available, which may contribute to a more timely achievement of effective therapeutic concentrations.

## Data Availability

The original contributions presented in the study are included in the article/Supplementary Material, further inquiries can be directed to the corresponding authors.
