# Supplementary material for: Precision dosing of voriconazole in immunocompromised children under 2 years: integrated machine learning and population pharmacokinetic modeling
Source: Front Pharmacol. 2025 Sep 15;16:1671652. doi: 10.3389/fphar.2025.1671652 (PMC12477134; doi:10.3389/fphar.2025.1671652)
Supplement: Supplementary file 1 [file DataSheet1.docx]

**Supplementary Table 1 The description of study samples**

| **Characteristic** | **Training set (n=77)** | **Validation set (n=33)** | **p value** |
| --- | --- | --- | --- |
| VRZ concentration, mg/L | 0.98 (0.34-2.05) | 1.2 (0.66-1.93) | 0.68 |
| Age, month | 13.00 (8.00-17.00) | 13.00 (9.00-15.00) | 0.73 |
| Gender, No. (%) |  |  | 0.96 |
| Male | 61 (79.22) | 26 (78.79) |  |
| Female | 16 (20.78) | 7 (21.21) |  |
| Weight, kg | 8.00 (6.30-9.30) | 8.50 (7.00-10.00) | 0.32 |
| Height, cm | 70.00 (66.00-75.00) | 71.00 (67.00-75.00) | 0.53 |
| BSA, m^2^ | 0.39 (0.39-0.44) | 0.41 (0.37-0.47) | 0.37 |
| Total daily dose, mg | 100.00 (100.00-133.20) | 132.00 (80.00-150.00) | 0.73 |
| CL, L/h/70kg | 3.18 (2.28-6.64) | 3.50 (2.30-5.51) | 0.31 |
| V, L/70kg | 90.07 (70.93-104.71) | 95.70 (78.81-112.59) | 0.32 |
| WBC, 10^^9^/L | 6.20 (4.15-10.00) | 6.45 (3.61-12.48) | 0.69 |
| N% | 58.20 (37.10-75.00) | 56.80 (32.30-67.80) | 0.44 |
| L% | 27.70 (10.40-46.40) | 30.40 (19.40-47.80) | 0.32 |
| RBC, 10^^12^/L | 3.66 (2.93-4.25) | 3.77 (3.39-4.10) | 0.43 |
| HGB, g/L | 105.00 (92.00-112.00) | 111.00 (97.00-120.00) | 0.13 |
| ALB, g/L | 36.50 (34.30-39.00) | 36.74 (34.06-40.56) | 0.52 |
| PLT, 10^^9^/L | 162.00 (55.00-425.00) | 137.00 (80.00-425.00) | 0.76 |
| CRP, mg/L | 0.62 (0.50-5.61) | 0.82 (0.50-4.00) | 0.82 |
| TBIL, μmol/L | 6.30 (3.30-11.70) | 6.90 (3.50-12.80) | 0.24 |
| DBIL, μmol/L | 2.80 (1.60-6.50) | 3.30 (1.70-6.80) | 0.27 |
| ALT, U/L | 39.40 (22.30-81.30) | 39.59 (23.82-95.74) | 0.26 |
| AST, U/L | 55.04 (40.35-89.35) | 57.93 (39.47-120.70) | 0.31 |
| ALP, U/L | 232.80 (124.00-267.00) | 205.52 (98.88-273.00) | 0.72 |
| SCR, μmol/L | 17.80 (13.90-21.00) | 18.00 (15.00-20.50) | 0.83 |
| EGFR, mL/min/1.73 m² | 150.39 (123.44-187.39) | 141.88 (128.74-178.24) | 0.75 |
| INR | 0.98 (0.91-1.06) | 0.95 (0.91-1.00) | 0.44 |
| D-Dimer, mg/L | 0.68 (0.50-0.92) | 0.65 (0.37-0.92) | 0.96 |
| Co-medication (n, %) |  |  |  |
| Tacrolimus | 33 (42.86) | 10 (30.30) | 0.22 |
| Sirolimus | 4 (5.19) | 1 (3.03) | 0.62 |
| Cyclosporine | 11 (14.29) | 8 (24.24) | 0.21 |
| Proton pump inhibitor | 44 (57.14) | 19 (57.58) | 0.97 |
| Glucocorticoid | 55 (71.43) | 26 (78.79) | 0.42 |

**Supplementary Table 2** Independent External Validation Population Characteristics

| **Characteristic** | **Mean ( SD )** | **Median** | **IQR** |
| --- | --- | --- | --- |
| No. of patients/samplings | 10/10 | | |
| Gender (Boys/Girls) | 7/3 | | |
| Age, months | 13.30 (8.5) | 14.00 | 5.50-21.50 |
| Height, cm | 70.45 (10.80) | 69.25 | 64.75-76.50 |
| Weight, kg | 7.93 (2.64) | 7.75 | 6.00-10.23 |
| BSA, m^2^ | 0.39 (0.09) | 0.38 | 0.33-0.46 |
| VRZ concentration, mg/L | 1.63 (1.41) | 1.25 | 0.66-2.58 |
| Therapy duration at TDM, days | 5.70 (1.06) | 5.50 | 5.00-6.75 |
| Total daily dose, mg | 112.46 (49.83) | 90.00 | 72.50-145.50 |
| WBC, 10^^9^/L | 7.74 (4.88) | 6.55 | 3.96-9.82 |
| N% | 38.22 (17.47) | 32.60 | 31.13-45.83 |
| L% | 45.18 (21.88) | 48.65 | 32.30-60.17 |
| RBC, 10^^12^/L | 3.52 (0.54) | 3.49 | 3.28-3.95 |
| HGB, g/L | 98.60(12.21) | 99.50 | 90.00-108.75 |
| ALB, g/L | 36.94 (4.13) | 37.48 | 34.58-39.62 |
| PLT, 10^^9^/L | 228.30 (173.18) | 173.50 | 109.50-340.00 |
| CRP, mg/L | 1.60 (1.84) | 0.72 | 0.50-2.38 |
| TBIL, μmol/L | 6.32 (4.13) | 5.65 | 3.58-6.60 |
| DBIL, μmol/L | 3.18 (2.08) | 2.75 | 1.70-3.38 |
| ALT, U/L | 41.51 (18.65) | 39.50 | 33.81-56.29 |
| AST, U/L | 51.33 (13.83) | 52.28 | 42.19-60.19 |
| ALP, U/L | 232.95 (116.11) | 248.44 | 139.69-271.25 |
| SCR, μmol/L | 16.88 (5.12) | 16.15 | 13.10-19.38 |
| EGFR, mL/min/1.73 m² | 174.91 (60.51) | 174.72 | 116.82-213.66 |
| INR | 0.97 (0.07) | 0.95 | 0.92-1.00 |
| D-Dimer, mg/L | 0.59 (0.31) | 0.57 | 0.30-0.89 |
| Co-medication (n, %) |  | | |
| Tacrolimus | 1 (10.00%) | | |
| Cyclosporine A | 3 (30.00%) | | |
| Proton pump inhibitor | 2 (20.00%) | | |
| Glucocorticoids | 4 (40.00%) | | |

**Supplementary Table 3.** Parameter estimates of voriconazole final PopPK model and bootstrap validation

| Parameter | Final model | | Bootstrap | |
| --- | --- | --- | --- | --- |
|  | Estimate | RSE% | Median | 95%CI |
| CL/F (L/h/70kg) | 17.9 | 10.8 | 17.65 | 9.11-22.00 |
| V/F (L/70kg) | 788 | 15.4 | 787.75 | 94.82-1050.85 |
| Ka (h-1) | 1.19 (fixed) | / | / | / |
| ω2CL/F | 0.674 | 10.7 | 0.672 | 0.175-0.976 |
| σ2 | 0.16 | 18.4 | 0.152 | 0.064-0.282 |

**Supplementary Table 4.** The evaluation of model performances on validation group

| Model Name | MSE | RMSE | MAE | R^2^ |
| --- | --- | --- | --- | --- |
| XGBoost | 0.28 | 0.53 | 0.40 | 0.81 |
| LightGBM | 0.46 | 0.68 | 0.50 | 0.69 |
| GBDT | 0.40 | 0.63 | 0.47 | 0.73 |
| CatBoost | 0.68 | 0.82 | 0.61 | 0.54 |
| AdaBoost | 0.41 | 0.64 | 0.48 | 0.72 |
| RF | 0.29 | 0.54 | 0.41 | 0.80 |
| Mean_scores | 0.42 | 0.64 | 0.48 | 0.72 |

**Abbreviations:** GBDT, gradient boosting decision tree; RF, random forest. MSE, mean squared error; RMSE, root mean squared error; MAE, mean absolute error.

**Supplementary Table 5** Performance metrics of the final XGBoost model across ten bootstrap cross-validation iterations

| Iteration | MSE | RMSE | MAE | R^2^ |
| --- | --- | --- | --- | --- |
| 1 | 0.3567 | 0.5972 | 0.4593 | 0.8000 |
| 2 | 0.3836 | 0.6193 | 0.4626 | 0.7891 |
| 3 | 0.3341 | 0.5780 | 0.4038 | 0.8199 |
| 4 | 0.2028 | 0.4503 | 0.3286 | 0.8674 |
| 5 | 0.3874 | 0.6224 | 0.4723 | 0.7968 |
| 6 | 0.3519 | 0.5932 | 0.4661 | 0.8170 |
| 7 | 0.3417 | 0.5845 | 0.4493 | 0.8108 |
| 8 | 0.4358 | 0.6601 | 0.4596 | 0.7792 |
| 9 | 0.3734 | 0.6111 | 0.4436 | 0.7757 |
| 10 | 0.3897 | 0.6243 | 0.4710 | 0.7935 |
| **Mean** | 0.3557 | 0.5940 | 0.4416 | 0.8049 |


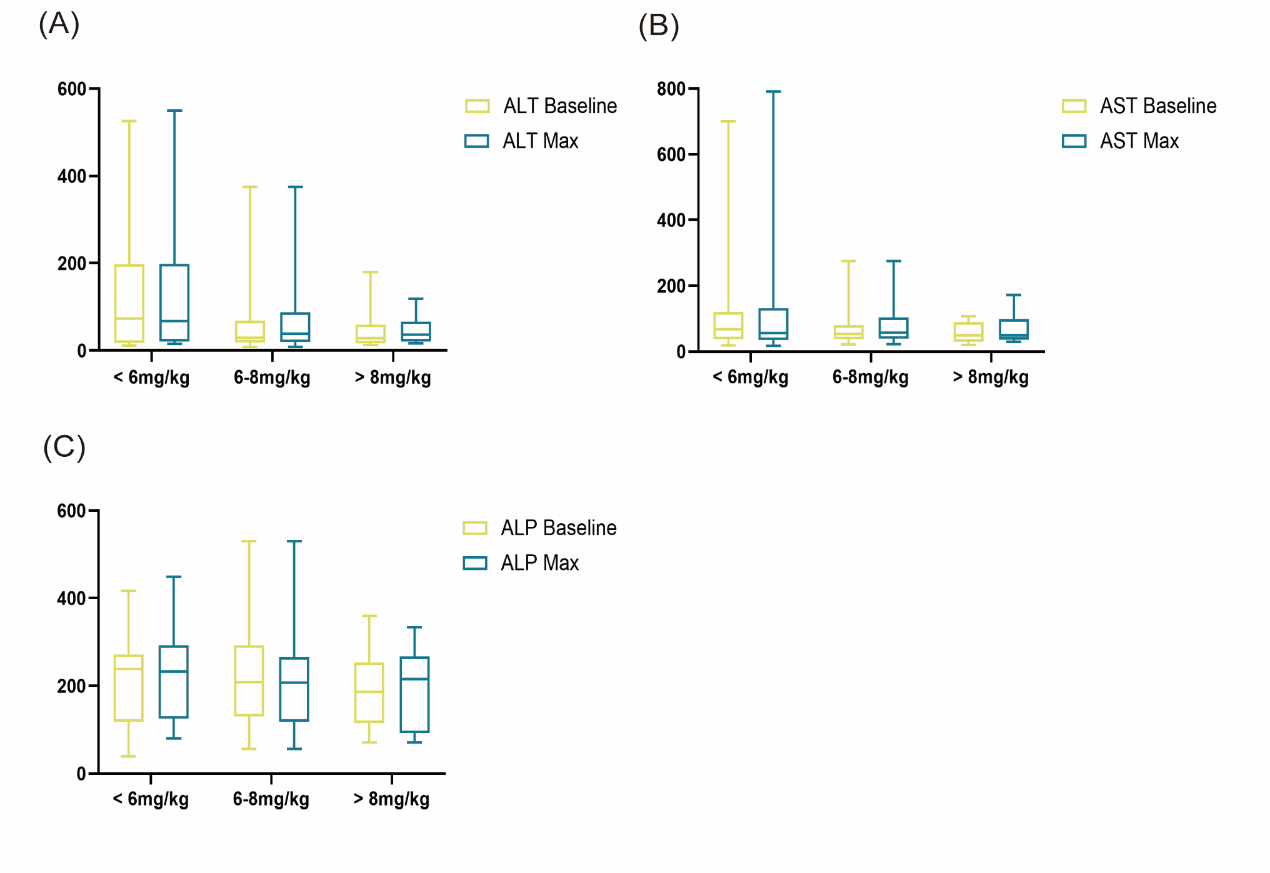


**Supplementary Figure 1** Parameters of Hepatic Function in Different Dosage Groups. This visual compared baseline ALT, AST, and ALP levels to maximum values during voriconazole therapy; Analysis of Variance showed no significant difference (p = 0.821, 0.064, and 0.372, respectively). ALT, alanine aminotransferase; AST, aspartate aminotransferase; ALP, alkaline phosphatase.


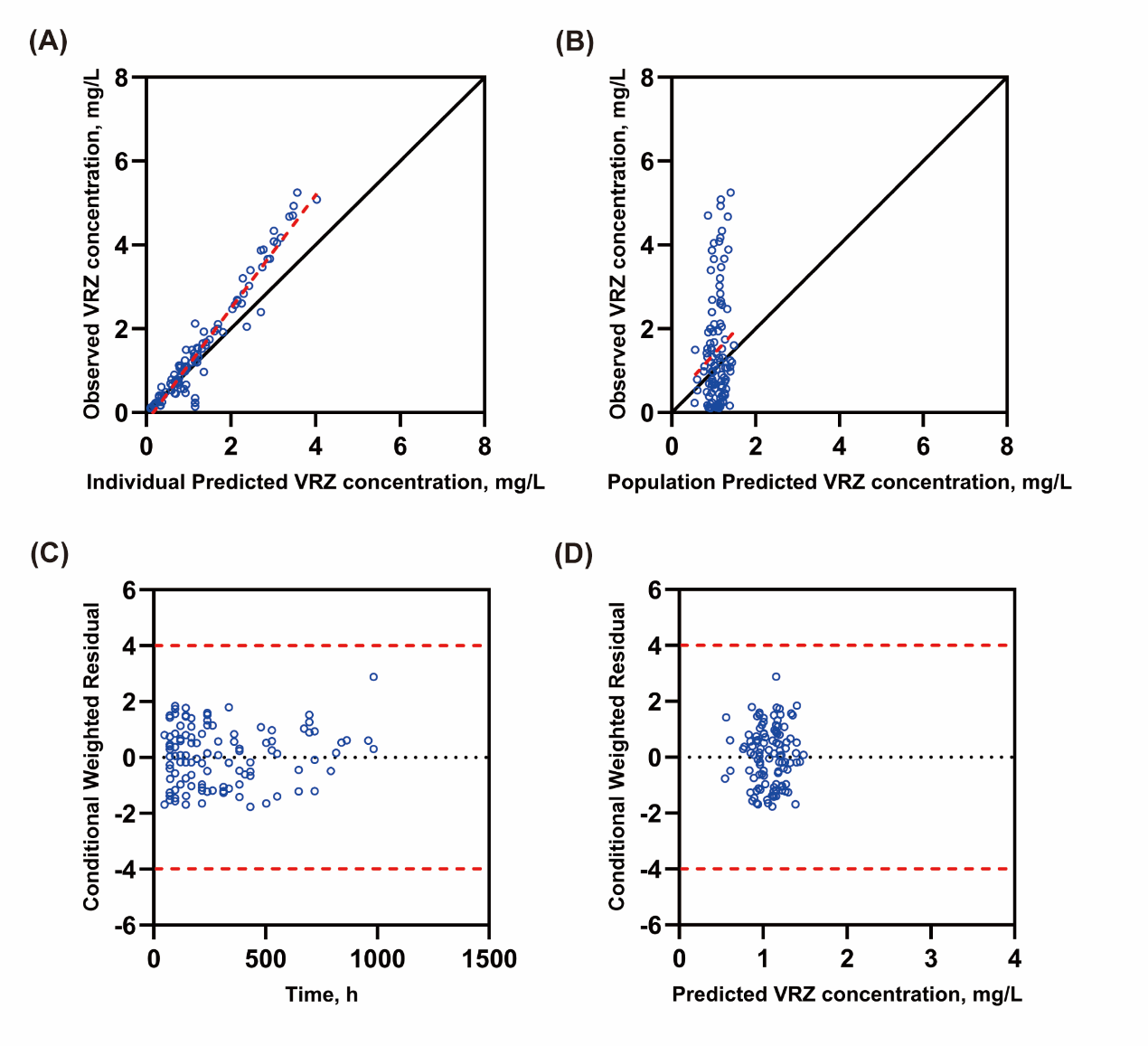


**Supplementary Figure 2** Goodness of fit model for Voriconazole. (A) Individual predicted VRZ concentration vs observed VRZ concentration. (B) Population predicted VRZ concentration vs observed VRZ concentration. (C) Analysis of conditional weighted residuals. (D) Predicted VRZ concentration vs conditional weighted residuals.


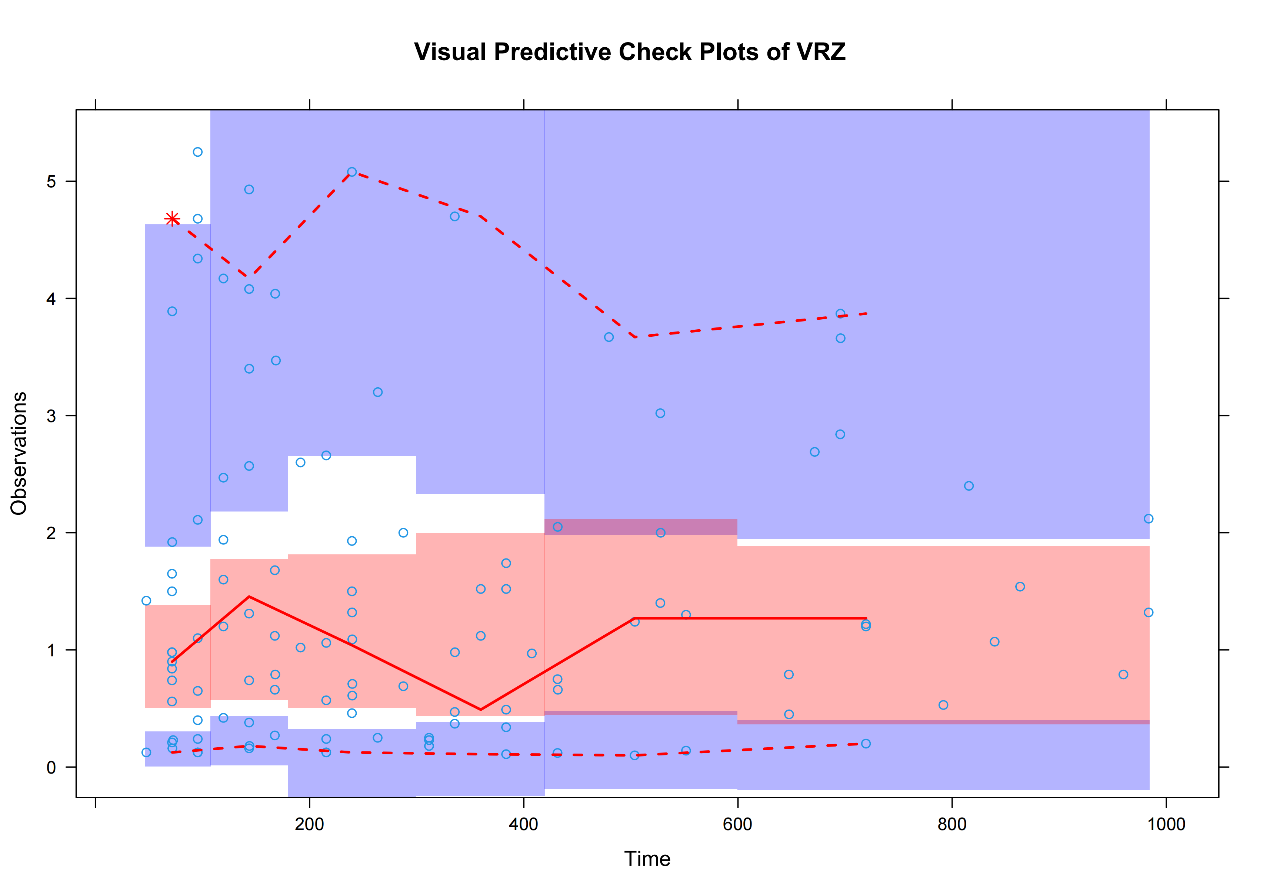


**Supplementary Figure 3** Visual Predictive Check Plots of Voriconazole
